# *In Vivo* Neuromechanics: Decoding Causal Motor Neuron Behavior with Resulting Musculoskeletal Function

**DOI:** 10.1038/s41598-017-13766-6

**Published:** 2017-10-18

**Authors:** Massimo Sartori, Utku Ş. Yavuz, Dario Farina

**Affiliations:** 1 Institute of Biomedical Technology and Technical Medicine, Department of Biomechanical Engineering, University of Twente, Enschede, The Netherlands; 2 Pain Medicine, Department of Anaesthesiology, University Medical Center Göttingen, Georg-August University, Göttingen, Germany; 3 Department of Bioengineering, Imperial College London, London, United Kingdom

## Abstract

Human motor function emerges from the interaction between the neuromuscular and the musculoskeletal systems. Despite the knowledge of the mechanisms underlying neural and mechanical functions, there is no relevant understanding of the neuro-mechanical interplay in the neuro-musculo-skeletal system. This currently represents the major challenge to the understanding of human movement. We address this challenge by proposing a paradigm for investigating spinal motor neuron contribution to skeletal joint mechanical function in the intact human *in vivo*. We employ multi-muscle spatial sampling and deconvolution of high-density fiber electrical activity to decode accurate α-motor neuron discharges across five lumbosacral segments in the human spinal cord. We use complete α-motor neuron discharge series to drive forward subject-specific models of the musculoskeletal system in open-loop with no corrective feedback. We perform validation tests where mechanical moments are estimated with no knowledge of reference data over unseen conditions. This enables accurate blinded estimation of ankle function purely from motor neuron information. Remarkably, this enables observing causal associations between spinal motor neuron activity and joint moment control. We provide a new class of neural data-driven musculoskeletal modeling formulations for bridging between movement neural and mechanical levels *in vivo* with implications for understanding motor physiology, pathology, and recovery.

## Introduction

Motor function in humans and animals is accomplished via neural control of muscle contractions that generate interaction forces throughout the skeletal system^1,2^. Motor function may be voluntary, take place at the level below awareness or, in the case of a reflex, it may bypass the brain^3^. It may emerge from a set of hardwired, in-born motor programmes^4^ or as a result of learning, refinement, and adaptation process^5–8^.

Motor function emerges from the neuro-mechanical interplay taking place in the composite neuro-musculo-skeletal system^2,9^. Spinal motor neurons are recruited to elicit mechanical forces that enable us to physically interact with the environment. Despite the extensive knowledge we have on neural microstructure function, we have little understanding of its causal association with the mechanical forces it provokes^10^. For example, the neural basis for accurate control of muscle fiber contraction and mechanical force steadiness are still debated^11,12^. Although musculoskeletal joint mechanical characteristics (i.e., moment^13^, loading^14^, stiffness^15,16^, damping^15^) are determined by underlying neuromuscular function^3,17^, establishing cause-effect relations between the neural and mechanical levels is an open challenge^18^. A central characteristic of motor control processes is state dependence. The mechanical function emerging from neural commands is dependent upon the states of the neuro-musculo-skeletal system. Even if it was possible to record neural function concurrently with the resulting mechanical function in a specific condition, this would only determine a correlation that would not be valid in a different condition (i.e. underlying different states), and would therefore be not causal^17^.

Neurophysiological analysis gives insights into the neural processing underlying human motor function, i.e. the generation of synaptic input ultimately converging to spinal motor neuron pools and recruitment of the innervated muscle fibers. This analysis alone, however, does not fully explain the mechanical significance and purpose of the observed neuromuscular mechanisms as it mostly focuses on the neural-side of the system not fully accounting for the form and function of musculoskeletal-side^1,2^.

The contribution of individual muscles to joint actuation has been previously studied by modeling a finite set of neuromuscular reflexes^19^ or by using *a priori* defined optimization criteria, e.g. minimal squared muscle activation sum or metabolic cost of transport. Modeling formulations based on networks of spiking motor, sensory, and inter-neurons were also recently used for explaining the control of single-joint musculoskeletal models during postural tasks^20,21^. However, an individual’s neuro-musculo-skeletal function (i.e., neuronal interaction, muscle fiber recruitment, musculoskeletal force production) highly varies across motor tasks^22–24^, pathology^25–28^, or training^29,30^, with variations occurring among individuals^31^ as well as within the same person^23,24^. Therefore, although current theoretical models provide a valuable starting point for the computational investigation of motor function, they are limited in reproducing an individual’s neuro-musculo-skeletal processes across different conditions (i.e. tasks, training, or pathologies). An alternative approach is thus needed.

Here we propose a paradigm to study human motor function at the interface between *in vivo* neurophysiology and biomechanics^10^. We propose a novel technique for probing the mechanical forces elicited by the activity of spinal motor neuron pools displaced across five lumbosacral segments of the human spinal cord. We apply this paradigm to a large repertoire of isometric ankle joint moment control tasks performed at different contraction efforts and joint angular positions. During these tasks, we decode spinal motor neuron activity and concurrently reconstruct the multi-muscle force patterns elicited in the control of ankle moments.

The ability of decoding the activity of large motor neuron pools potentially provides access to the synaptic input converging onto these pools^32^, thus opening new avenues for understanding the organization and connections in higher spinal neural networks. The breakthrough with respect to current theoretical models (not driven by neural signals) is that our proposed approach enables decoding subject-specific motor neuron strategies across any condition (i.e. task, pathology, training) from muscle fiber electrical activity, with no direct need for creating numerical models of spinal neuronal networks. Importantly, this is done in a purely open-loop way, i.e. with no closed-loop corrective mechanism that compensates for moment prediction errors. In our proposed formulation, joint moment estimates are therefore purely contributed by *in vivo* motor neuron activity. This opens new avenues for understanding neuro-mechanical causalities in human movement and the alterations caused by impairment^33^, with potentials for establishing novel treatments.

## Results

**Figure 1:**
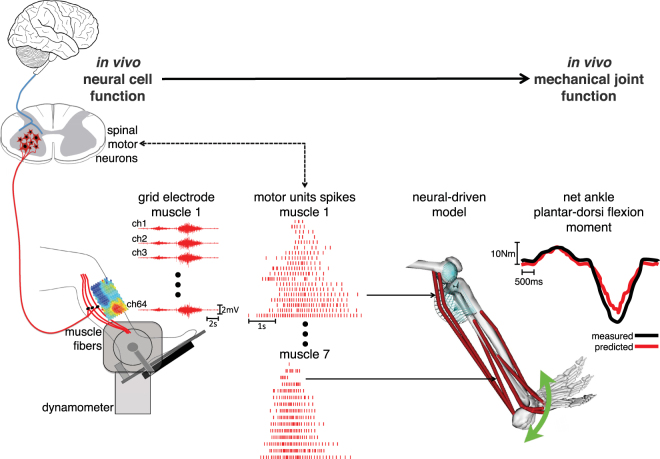
Linking between the neural and mechanical levels of motor function. Subjects perform a large repertoire of isometric ankle plantar-dorsi flexion contractions. High-density electromyograms are recorded and decomposed into the temporal events at which the underlying motor neurons discharged, thus opening a window into the central nervous system. This is done for seven major muscles spanning the ankle joint (see Methods Section). The decoded motor neuron discharges are directly used to control a subject-specific model of the musculoskeletal system. This enables reconstructing the net ankle moment over time, without knowledge of the experimental values (i.e. blinded validation) and without closed-loop feedback mechanism for compensating prediction errors (i.e. open-loop formulations). In this way, the predicted moment is purely contributed by motor neuron-controlled multi-muscle contractions acting on the skeletal system.

**Figure 2:**
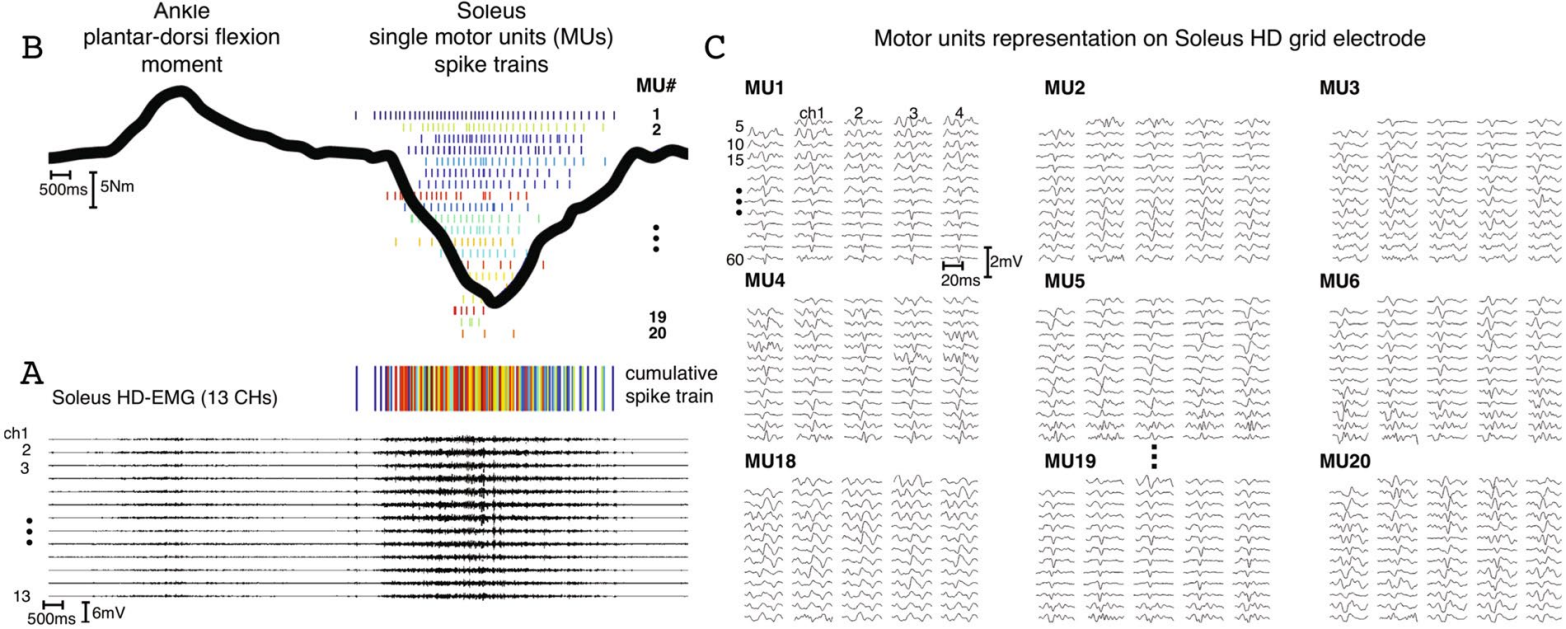
Separating the interferent electromyogram into its central and peripheral components. (**A**) High-density electromyograms (HD-EMGs) visualized for 13 out of 64 channels located in the middle column of a 13 × 5 electrode grid. This signal is recorded from the soleus muscle during a 30% maximal voluntary contraction performed with ankle joint at neutral position. (**B**) Experimental ankle plantar-dorsi flexion moment (continuous curve) depicted synchronously with soleus HD-EMG (**A**) and the decoded motor neuron discharge events (spike trains). (**C**) The interferent HD-EMG (**A**) is decomposed into motor unit (MU) action potentials (peripheral component) and motor neuron spike trains (central component, discrete spikes in **B**). Spike trains are combined together to form the cumulative spike train, i.e. an accurate estimate of the net neural drive produced by the nervous system in the control of a muscle. The decoded neural drive is subsequently used as part of the neural data-driven modeling formulation (Figs 3–5).

**Figure 3:**
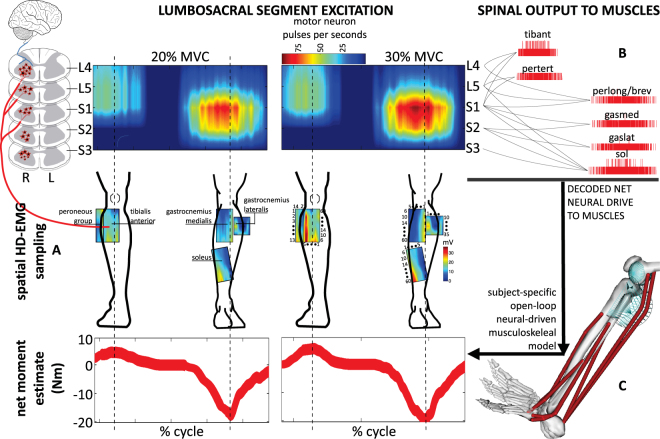
Spatiotemporal patterns of ipsilateral α-motor neuron activity in the spinal cord. (**A**) Multi-muscle spatial sampling of high-density electromyograms (HD-EMGs) provides access to high-dimensional data-streams of muscle fiber electrical activity via 256 EMG channels simultaneously. Colormaps represent the HD-EMG root mean square value over the electrodes of the grids used for recording. (**B**) The HD-EMG is decomposed to determine cumulative spike trains of the motor neurons innervating selected musculotendon units, including tibialis anterior (tibant), peroneus tertius (pertert), brevis (perbrev), and longus (perlong), gastrocnemius lateralis (gaslat) and medialis (gasmed), and soleus (sol). The instantaneous discharge rates (pulses per seconds) of the cumulative spike trains are mapped onto the rostrocaudal axis of the spinal cord lumbosacral segments, i.e. L4–5 and S1–3. The multiple spinal segments innervating a single muscle are visualized via curved lines connecting a lumbosacral segment to the resulting cumulative spike train to the muscle. The α-motor neuron-dependent lumbosacral segments activity is visualized using a color scale via a filled contour plot (Methods Section). (**C**) Motor neuron spike trains drive forward an open-loop subject-specific musculoskeletal model. This reveals how the spinal cord output layers generate the neural drive to muscles to enable ankle joint mechanical moment control across different forces (%MVC).

**Figure 4:**
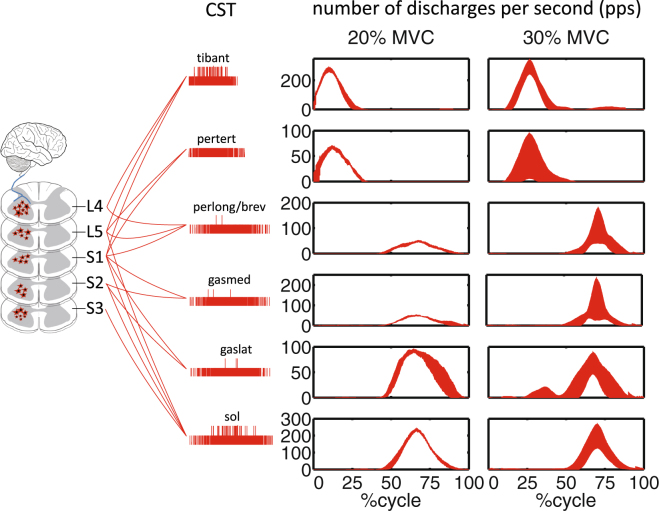
The neural drive to muscles. Total number of pulses per second (pps) discharged by the population of motor neurons decoded across two levels of percentage maximal voluntary contraction (%MVCs). Discharge profiles are computed from motor neuron cumulative spikes trains (CST, Eq. 1) for all investigated muscles including: tibialis anterior (tibant), peroneus tertius (pertert), peroneus longus and brevis (perlong), soleus (sol), gastrocnemius lateralis (gaslat) and gastrocnemius medialis (gasmed). The 0% denotes the onset of the dorsi-flexing phase while the 100% denotes the completion of the plantar-flexing phase of the task. Data are reported for the ankle anatomical angular position. Within each graph values are averaged across all validation trials and subjects, with depicted profiles reflecting the first standard deviation band.

**Figure 5:**
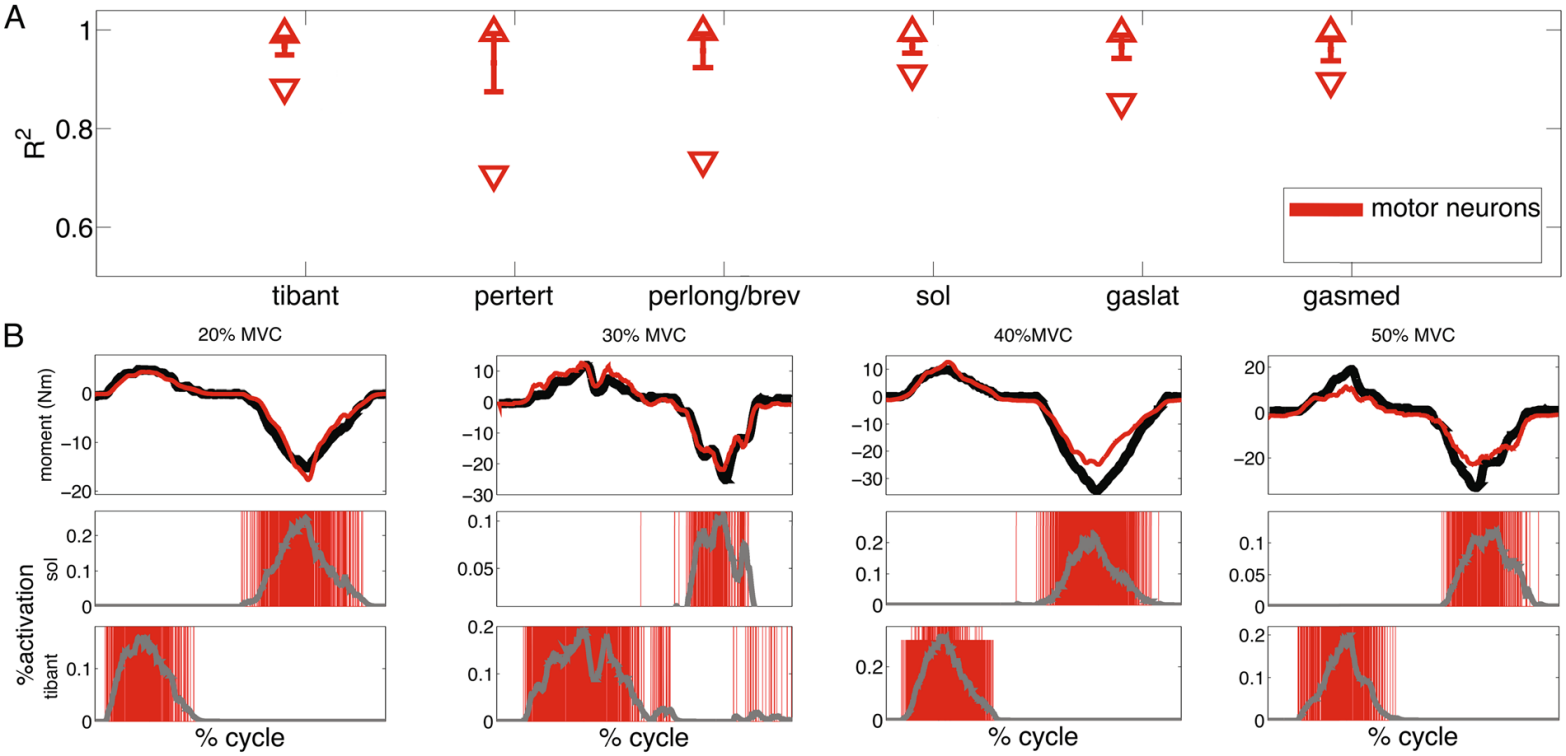
Normalized muscle activation and joint moment profiles. (**A**) Square of the Pearson product moment correlation coefficient (R^2^) between reference ankle joint moments and activation profiles derived from motor neuron cumulative spike trains (Eq. 3, see Methods Section). Activation profiles are normalized to vary between 0 (i.e. no muscle recruitment) and 1 (i.e. maximal muscle recruitment, Methods Section). Similarity is computed during the dorsi-flexion phase for the tibialis anterior (tibant) and peroneus tertius (pertert) and during the plantar-flexion phase for the peroneus longus, peroneus brevis (perlong), soleus (sol), gastrocnemius lateralis (gaslat) and gastrocnemius medialis (gasmed). Downwards and upwards triangles represent R^2^ absolute minimal and maximal values respectively. Vertical lines represent R^2^ standard deviation ranges. Values are derived across all trials and subjects. (**B**) Representative joint moment profiles including reference moments (black curve) and motor neuron-contributed moment (red curve). Second and third row graphs report tibant and sol motor neuron cumulative spike trains (red vertical lines) and resulting neural activation profiles (grey curves, Eq. 3) highlighting that neural activation shape is well mimicked by net reference moment shape. The 0% denotes the onset of the dorsi-flexing phase while the 100% denotes the completion of the plantar-flexing phase of the task.

**Figure 6:**
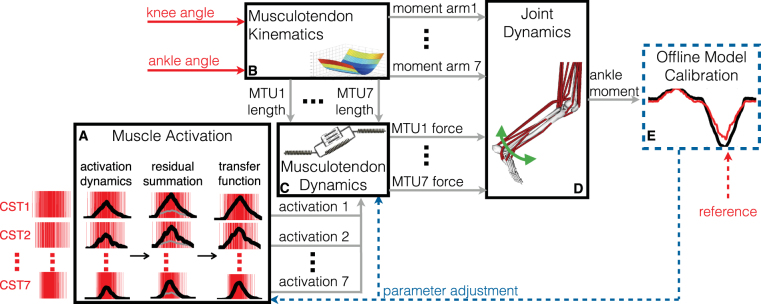
Schematics of the neural data-driven musculoskeletal modeling formulation. The model (**A**–**D**) is initially calibrated offline (**E**). Then, it takes experimental knee and ankle joint angles and spinal motor neuron cumulative spike trains (CSTs) as input and computes all transformations that lead to musculotendon force and ankle joint moment production. The muscle activation component (**A**) converts motor neuron CSTs into neural activation/deactivation profiles using a second order recursive system (Eq. 3). This is summed to the residual interferent electromyogram component not decomposed into CSTs (see Methods Section) to recover the muscle twitch response and further processed via a nonlinear transfer function to compute the resulting muscle activation (Eq. 4). The musculotendon kinematics component (**B**) synthetizes subject-specific three-dimensional paths of musculotendon units (MTUs) using a set of MTU-specific multidimensional cubic B-splines. Each B-spline computes MTU length and moment arms as a function of the input joint angles. The MTU dynamics component (**C**) solves for the dynamic equilibrium between muscle fibers and series elastic tendons in the production of net MTU force. It employs a Hill-type muscle model informed by MTU length and muscle activations from the previous two components. The joint dynamics component (**D**) transfers MTU forces to the skeletal joint level using MTU moment arms. In the offline calibration component (**E**), initial nominal parameters are repeatedly refined, as part of a least-squares optimization procedure, so that the mismatch between model’s predicted joint function and the experimentally recorded joint function is minimized. After calibration, the entire estimation procedure runs in open-loop without measurement-driven error compensation.

**Figure 7:**
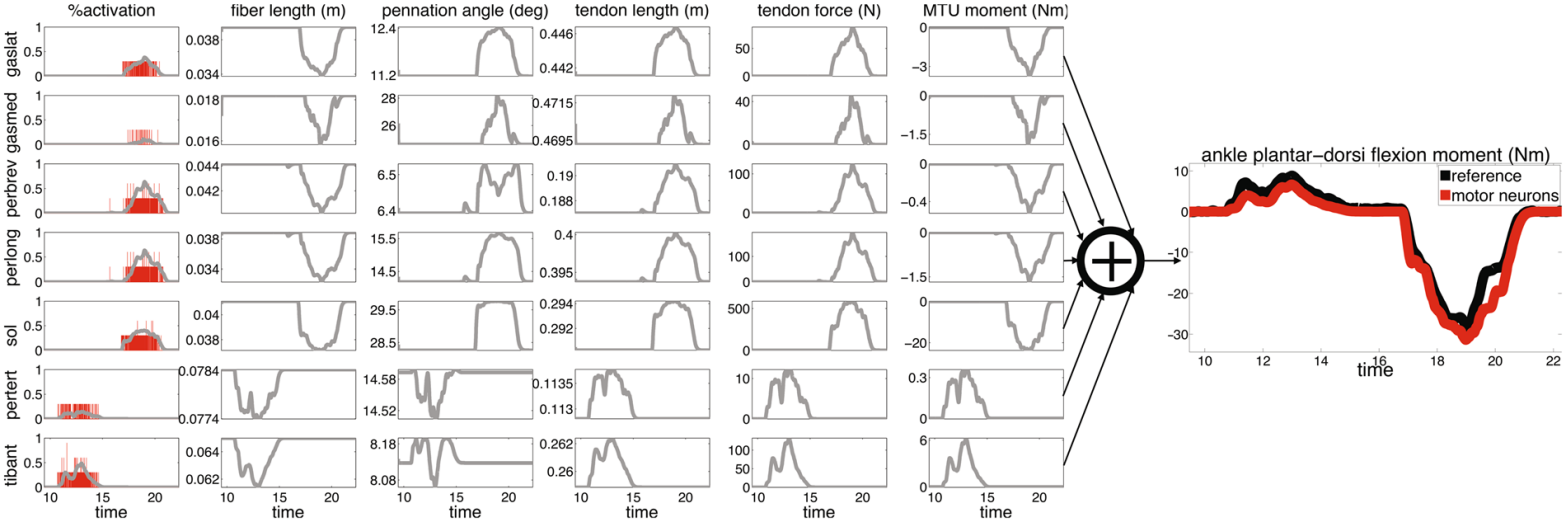
The neuro-mechanical processes underlying joint moment control. Open-loop translation of input motor neuron cumulative spike trains (CSTs) into output joint moment. This involves the conversion of discrete CSTs (red vertical lines in left-hand graphs) into continuous muscle activation profiles (Eq. 4), which are then used to determine time-varying fiber lengths, pennation angles, tendon lengths, net musculotendon unit (MTU) forces and moments. MTU-specific moments produced by the coordinated multi-MTU function are summed up to compute the resulting net moments contributed about the ankle plantar-dorsi flexion degree of freedom. The CST-derived joint moment (red curve) is compared for validation with reference values (black curve) derived from dynamometer readings. The neuro-mechanical steps are reported for all modeled MTUs: tibialis anterior (tibant), peroneus tertius (pertert), peroneus longus (perlong), peroneus brevis (perlprev), soleus (sol), gastrocnemius lateralis (gaslat) and gastrocnemius medialis (gasmed). Data are depicted for a representative trial performed at 30% of maximal voluntary contraction (%MVC) with the ankle join at 10 degrees of dorsi-flexion.

**Figure 8:**
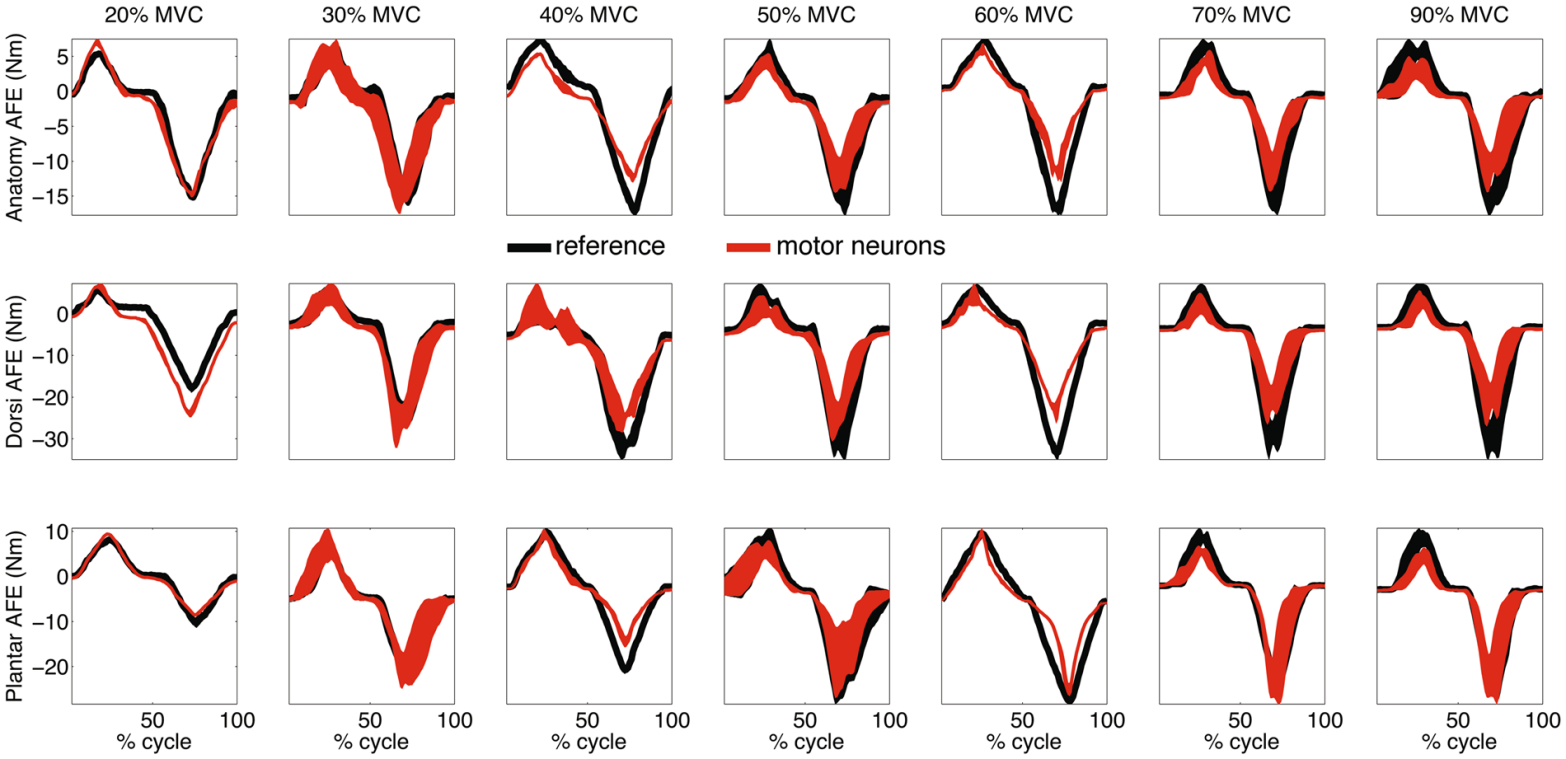
Predicted and reference joint moments. Net ankle plantar-dorsi flexion (AFE) moments performed at different percentages of maximal voluntary contraction (%MVC) forces and at three angular positions including the anatomical position (Anatomy), 10 degrees dorsi-flexion (Dorsi) and 10 degrees plantar-flexion (Plantar). The reported joint moment profiles are the reference dynamometer readings (black curve) and those predicted using motor neuron spike trains (red curve). These are relative to the first band of standard deviation computed from all 207 validation trials performed by all subjects. The 0% denotes the onset of the dorsi-flexing phase while the 100% denotes the completion of the plantar-flexing phase of the task.

Subjects seated on a dynamometer and were instructed to perform isometric ankle plantar-dorsi flexion contractions at seven levels of maximal voluntary contraction (%MVCs) repeated at three ankle joint angles (Fig. 1, Methods Section). We established an interface with the human nervous system by employing multi-muscle spatial sampling of high-density muscle fiber electromyograms (HD-EMG, Figs.1–2). This involved more than 250 recording sites over seven leg muscles (Methods Section). We decomposed the muscle fiber HD-EMG to open a window into α-motor neuron activity and to its distribution across the rostrocaudal axis of the spinal cord (Figs 3–5). We then established a neural data-driven musculoskeletal model that was scaled and calibrated to each subject (Fig. 6, Methods Section)^34^. The subject-specific model was validated on the ability of translating motor neuron discharges into the resulting net ankle joint moments (Figs 7,8, Supplementary Video S1)^34^. This was done in an open-loop way (i.e. no closed-loop corrective mechanism) on unseen trials that were not used for calibrating the model internal parameters (Fig. 6E, Methods Section). Results showed that the proposed formulation could synthetize realistic neuro-mechanical processes (i.e. highly non-linear) involved in the joint moment control task (Fig. 7, Supplementary Video S1). Remarkably, results also showed that our proposed paradigm enabled revealing direct associations between modulations in spinal motor neuron activity and joint moment control (Figs 5 and 8). This confirmed the possibility of observing the link existing between the neural and mechanical function *in vivo* in the intact human. This Section presents quantitative results across all stages of the analyses.

### Decoding the net neural drive to muscles

Figure 2 depicts soleus muscle fibers HD-EMGs recorded during a 30% MVC trial performed at anatomical ankle angular position. The figure shows how the interferent HD-EMG was decomposed into its neural (i.e., motor neuron discharges) and peripheral components (i.e., muscle fiber action potentials). In this depicted trial, we could decode a total of 20 motor neurons active between 48% and 89% of the trial cycle. For all subjects and trials, the proposed sampling and decoding method always detected on average >10 motor neurons per muscle. The largest motor neuron populations were decoded from the soleus (13 ≤ size ≤ 29) and tibialis anterior (12 ≤ size ≤ 26). Smaller populations were decoded from the gastrocnemius medialis (3 ≤ size ≤ 17), gastrocnemius lateralis (2 ≤ size ≤ 12) and peroneus group (5 ≤ size ≤ 14). Single motor neuron discharge rates (DRs, Eq. 1) were always within physiological ranges, i.e. ≤ 44.4 pulses per seconds (pps) in all conditions^35^. Absolute ranges of variations (i.e. highest and lowest observed rates for a single motor neuron) were similar for single motor neurons controlling the tibialis anterior (3.3 ≤ DR ≤ 40.3 pps), soleus (3.9 ≤ DR ≤ 40.1 pps), peroneus group (5.9 ≤ DR ≤ 42.7 pps) and gastrocnemius medialis (4.6 ≤ DR ≤ 44.3 pps) whereas the lower bound for DR was greater for the gastrocnemius lateralis (9.7 ≤ DR ≤ 44.4 pps).

Figure 2A,B shows how single motor neuron spike trains were combined to form the cumulative spike train. This was used to compute the total motor neuron firing, i.e. the total number of discharges over time from the decoded population of α-motor neurons innervating a muscle, providing a direct indication of neural activity in the spinal cord. As detailed in the Methods Section (Eq. 2), we mapped total motor neuron firings onto the lumbosacral segments of the human spinal cord along the rostrocaudal axis. Figure 3 visualizes ipsilateral spinal segments activity in the transition from 20% to 30%MVC. During the plantar-flexion part of the task, spinal cord sacral segment activity increased from 96.4 pps (20% MVC) to 106.3 pps (30% MVC, Fig. 3). During the dorsi-flexion part of the task, spinal cord activity was substantial in the lumbar segments increasing from 57.6 pps (20% MVC) to 70.6 pps (30% MVC, Fig. 3).

The total motor neuron firing provided a direct estimate of the net neural drive produced by the nervous system in the control of a muscle, i.e. the ultimate neural code transduced by musculotendon units into mechanical force. We observed substantial modulations in this measure across muscles and conditions. In the transition from 20% to 90%MVC, the total motor neuron firing increased from 278.6 ± 95.6 pps to 374.4 ± 118.5 pps for the tibialis anterior, 34.6 ± 8.9 pps to 42.2 ± 34.1 pps for the peroneus tertius, 38.6 ± 5.8 pps to 214.8 ± 123.5 pps for the peroneus longus/brevis, 92.4 ± 17.7 pps to 122.7 ± 113.4 pps for the gastrocnemius lateralis, 44.3 ± 8.0 pps to 184.6 ± 122.1.6 pps for the gastrocnemius medialis, and 208.0 ± 19.9 pps to 268.9 ± 99.2 pps for the soleus. Figure 4 shows total motor neuron firing profiles for each muscle, averaged across all subjects in the transition from 20% to 30%MVC.

The discharge patterns we detected were highly accurate for all reported motor neurons as demonstrated by the threshold we imposed on the pulse-to-noise ratio (PNR) metrics in the HD-EMG decomposition (Methods Section)^36^. The PNR was always ≥0.9 for all the decoded motor neurons and contraction levels, thus assuring decomposition reliability, as we previously demonstrated^36^. This showed that we could detect accurate modulations of the net neural drive to muscles across force levels and joint angles. Whether the derived neural drive estimates were biomechanically relevant was addressed in the next two Sections.

### Associations between spinal motor neuron and joint mechanical function

Given the strong neuro-mechanical interplay in movement generation, we assessed whether we could reveal direct associations between modulations in decoded motor neuron activity and experimental joint moments^37^. This analysis further proved the reliability of the decoded spinal motor neuron activity (as analyzed in the previous section; PNR ≥ 0.9) and supported its biomechanical relevance.

Results showed that across all subjects, angles and %MVCs, the modulation of total motor neuron firing was a predominant mechanism of net joint moment control. Shape modulations in time-varying experimental moments mimicked those observed in neural activations derived from cumulative spike trains (Eq. 3, Fig. 5). We used the coefficient of determination R^2^ to compute shape similarity across all 207 validation trials (min ≤ mean ± std ≤ max) between net ankle dorsi-flexion moment and the neural activation generated by the motor neuron cumulative spike train produced in the control of each muscle. This was high for the dorsi-flexing muscles (Fig. 5), including the tibialis anterior (0.88 ≤ 0.97 ± 0.02 ≤ 0.99) and the peroneus tertius (0.71 ≤ 0.93 ± 0.06 ≤ 0.99). Likewise, theR^2^ between net ankle plantar-flexion moment and motor neuron activation was high for all plantar-flexing muscles (Fig. 5), including the soleus (0.91 ≤ 0.97 ± 0.01 ≤ 0.99), peroneus longus/brevis (0.74 ≤ 0.96 ± 0.03 ≤ 0.99), gastrocnemius medialis (0.89 ≤ 0.96 ± 0.02 ≤ 0.99) and lateralis (0.85 ≤ 0.97 ± 0.02 ≤ 0.99). A visual representation of these mechanisms is depicted in Fig. 5B and in Fig. 7, where distinctive joint moment modulations, even those particularly rapid (i.e., see 30% MVC condition in Fig. 5B), were directly represented in neural activation profiles.

### Predicting net joint moment from the neural drive to muscles

We then assessed whether our proposed neural data-driven modeling method (Fig. 6) could translate discrete cumulative spike trains derived from high-dimensional sets of motor neurons (on average 56.7 ± 10.2 across all muscles) into multi-muscle coordinated force profiles that could reconstruct experimental joint moments over all subjects and conditions in a blinded open-loop way. Figure 7 shows the individual non-linear neuro-mechanical transformations underlying net joint moment estimates as computed by the proposed method, i.e. from motor neuron discharges to modulation of musculotendon kinematics (i.e. fiber and tendon length), from coordinated multi-muscle moments to net ankle moment generation. Supplementary Video S1 further visualizes how input motor neuron spike trains were translated into output musculotendon force for the soleus muscle during a 20% MVC task at 10 degrees of ankle dorsi-flexion. The net joint moments could be well estimated directly from motor neuron discharges across a total of 207 validation trials performed by all subjects across the tested conditions (51.7 ± 5.6 validation trials per subject, Fig. 8, Methods Section). The computed similarity metrics between predicted and experimental moments were the coefficient of determination R^2^, the root mean squared error normalized to the root mean squared sum of experimental values (NRMSE), as well as the Chebyshev 90% confidence intervals for both R^2^ and NRMSE (Methods Section). These metrics ranged between 0.13 ≤ NRMSE ≤ 0.58 and 0.82 ≤ R^2^ ≤ 0.99 with Chebyshev maximal expected errors represented by R^2^ = 0.88 and NMRSE = 0.57 (Fig. 8). Of the 207 performed estimates, 97% had R^2^ > 0.9 and 87% had NRMSEs < 0.4, across all tasks and subjects (Fig. 8). The average values were distributed toward low-range NRMSEs (i.e., 0.3 ± 0.02) and high-range R^2^ (i.e., 0.96 ± 0.03) values (Figs 5B and 8).

Results confirmed that the proposed method was superior at predicting time-varying joint moment shape modulations than the most advanced methods available to date based on interferent high-density EMGs recorded using bi-dimensional grids of electrodes (Fig. S1). The R^2^ between experimental moment and the moment contributed by motor neuron discharges ranged between 0.87 ≤ R^2^ ≤ 1, with average values of R^2^ = 0.97 ± 0.01. When joint moments were predicted using linear envelopes derived from the interferent HD-EMGs, similarity ranged between less favorable values 0.67 ≤ R^2^ ≤ 0.98, with consistently lower average values of R^2^ = 0.92 ± 0.05 (Fig. S1). The information on motor neuron activity extracted from HD-EMG deconvolution enable to causally bridge neural activation to mechanical function, which was our primary hypothesis, and proved to be superior to the use of global HD-EMG features.

Overall, results confirmed that the proposed paradigm (HD-EMG spatial sampling, motor neuron decoding and neuro-mechanical modeling) enabled probing the *in vivo* mechanical forces actuating biological joints as a function of sampled spinal motor neuron pools, despite these pools representing only a subset of all active motor neurons.

## Discussion

We presented a paradigm to study human motor function at the interface between neurophysiology and biomechanics. This reveals how spinal motor neurons elicit mechanical forces at the musculoskeletal level *in vivo* in the intact human. It comprises two main components. The first is a neural interface that provides access to α-motor neuron activity across the spinal cord lumbosacral segments (Fig. 3). This is realized via multi-muscle spatial sampling and deconvolution of the electrical activity of muscle fibers innervated by α-motor neuron axons. The second is a subject-specific neural data-driven musculoskeletal modeling formulation. This enables translating *in vivo* motor neuron discharges and residual background activity into accurate estimates of the resulting mechanical function elicited in human biological joints.

We demonstrated the possibility of decoding hundreds of motor neuron discharges (Fig. 4) from multiple muscles concurrently and use them, in a biomechanically meaningful way, for the accurate computation of resulting joint moments (Figs 7–8) across an extensive repertoire of muscle contractile conditions, i.e. seven %MVCs repeated at three joint angles. Importantly, this was done purely in an open-loop way, i.e. motor neuron discharges were blindly converted into joint moments with no closed-loop mechanism that compensated for prediction errors. The remarkable aspect is that the approach revealed a direct causal association between modulations in motor neuron activity and modulations in joint moments (Figs 3, 5 and 7). This is an important mechanism at the basis of human and animal motor function^5,37,38^, which emerged from our proposed neural data-driven modelling formulation, thus supporting the validity of the approach. Accurate open-loop moment estimates across all conditions and subjects (over 207 validation trials) could only be obtained if the underlying neuro-mechanical modelling transformations (i.e. multi-muscle HD-EMG sampling, discharge decoding, multi-muscle coordination, net joint moment production, Fig. 7, Supplementary Video S1) well reflected the actual physiological processes of each subject. This achievement required addressing challenges, including the concurrent recording of large HD-EMG data streams (>250 channels) simultaneously from multiple muscles and the accurate separation of the interferent fiber electrical activity into its neural (motor neuron discharges) and its peripheral component (muscle fibers action potentials) as well as the creation of physiologically accurate neural data-driven musculoskeletal formulations that captured the subject-specific anthropometry and non-linear transformations between neural input and mechanical force output.

The innovative focus of the proposed procedure is the ability of combining high-density EMG spatial sampling, decoding and modeling in a single formulation. This represents a novel way to process interferent HD-EMG signals and obtain accurate estimates of the neuro-mechanical information it contains, i.e. causal motor neuron firing patters linked to mechanical forces. Furthermore, this offers a paradigm shift in the current field of musculoskeletal modeling^34^. Due to the indirect way in which the interferent bipolar EMG relates to muscle force generation and due to the difficulties in interpreting it for large muscles, current approaches cannot reveal the neural determinants of mechanical force production, a fundamental problem our proposed paradigm directly addresses. Current modeling formulations define neural activation to muscles as the intensity of single-channel EMG linear envelopes, i.e. differential signal from a bipolar electrode pair, rectified, filtered, and amplitude-normalized^3^. Because the interferent EMG is generated by the spatio-temporal convolution of thousands of motor neuron discharges, the temporal and spatial resolution of the linear envelope is described by a random process, which may introduce discrepancies with its physiological counterpart, i.e. this process is inherently biased by the transformation of a frequency-domain feature (i.e., motor neuron firing) into an amplitude-domain feature (i.e., linearly filtered rectified EMG intensity). In contrast, motor neuron discharges provide unbiased information, i.e. they reflect the exact physiological process underlying muscle activation and the actual way mechanical function is coded and modulated at the spinal level, as visualized in Fig. 3. The innovative focus is that, within our formulation, muscles become biological amplifiers of the spinal neural output and musculoskeletal modelling becomes the dynamical process that translates decoded neural discharges into mechanical force.

Results showed that the decoding of motor neuron discharges was accurate as determined by performance metrics computed during the decomposition process (PNR ≥ 0.9). This enabled accessing a large number of motor neurons (on average 56.7 ± 10.2 decoded neurons across seven muscles), which displayed a variety of firing behaviors across muscles and %MVCs (Figs 3–5). This also provided a challenging environment for testing whether the neural data-driven model could operate as a function of the diverse motor neuron firing trends. Results showed that the proposed neural-data driven modeling method could consistently translate the diverse motor neuron firing behaviors into accurate predictions of net ankle moment (Fig. 8). Remarkably, this was possible during 15 unseen %MVCs conditions (i.e. not used for model parameter identification, see Methods Sections), which demonstrated the ability of extrapolating beyond calibration conditions. This proves that the approach truly synthetized the underlying neuro-mechanical transformations and did not merely learn to fit input to output data in a specific instance. This gives confidence that the sampling and decoding can extract motor neuron populations that are biomechanically relevant despite representing only a part of the actual pool recruited in the human and that the proposed neural data-driven model provides a realistic representation of each subject’s neuro-musculo-skeletal function (Fig. 5). Our results showed the superiority of the proposed paradigm in predicting joint moment fine modulations with respect to the current state of the art methods based on interferent EMGs. The shape of net ankle plantar-dorsi flexion moment directly mimicked the shape of motor neuron-dependent neural activation profiles across all conditions, including those underlying particularly fast moment modulations (Fig. 5). Overall, results proved that our proposed neuro-mechanical modeling approach enabled deriving estimates of the mechanical forces elicited in multiple muscles by large pools of motor neurons with the highest fidelity (Figs 5, 7 and 8).

Future work will investigate whether our proposed methodology can extrapolate, not only across unseen %MVC conditions (Fig. 8), but also across time scales. Current traditional methods based on EMG linear envelopes are affected by changes in underlying action potential shape and amplitude across experimental sessions (i.e. due to electrode placement, electrode-to-skin impedance, etc.), thereby inducing variability in the overall EMG envelope. The ability of decomposing the EMG into motor neuron discharges has the potential to relax these constraints, i.e. the underlying spike trains are dimensionless and likely to be less affected by electrode-induced biases, provided that a sufficiently number of motor neurons is decoded. This may open the way to neuro-musculo-skeletal models that need to be scaled and calibrated once for all per subject. Our results showed the ability of linking motor neuron discharges and resulting joint moments during high-effort tasks (i.e. 70–90%MV) underlying fast (i.e. near-ballistic) muscle contraction. Future work will investigate the ability of translating the current approach to motor tasks that underlie highly dynamic contractions and a larger repertoire of muscle short-stretch cycles, i.e. including locomotion with muscle fibers operating eccentrically and concentrically over larger ranges of their force-length-velocity dependency.

This study also showed that the effective part of the cumulative spike train responsible for force modulation was in the low frequency band. This was represented via slow neural activation profiles, i.e., motor neuron spike trains filtered via a second-order twitch model (Eq. 3, Figs 5 and 7). The direct association found between neural activation and joint moment (Fig. 5) is explained by the fact that the musculoskeletal system acts as a natural low pass filter of the spinal segments neural output (Figs 3 and 4), a central mechanism that was properly captured by our paradigm (Fig. 7). The neural drive high frequency band is filtered out by the slow twitch response of muscle fibers triggering electrochemical transformations (i.e., calcium dynamic) and inducing limits in fiber action potential propagation velocity as well as by intrinsic viscoelasticity properties of muscle–tendon units. Previous studies have shown slow activation profiles to directly mirror the common synaptic input (i.e. common drive) projected from higher spinal neural levels down to alpha motor neuron pools^32,39^. In light of our results, this would suggest the common drive to be a prime neural mechanism in the modulation of mechanical function across several contraction efforts spanning both functional (i.e., 20% to 50%MVC in our study) and near-maximal contraction ranges (i.e., 90% MVC in our study).

This work was based on four subjects. Therefore, results may not be completely generalizable. However, this study aimed at developing the theoretical, experimental, and computational framework for investigating the neuro-mechanical interplay that regulates motor function in the intact human *in vivo*. This step needs to be necessarily taken before the framework can be systematically applied to a large population of subjects. Moreover, our proposed formulation enables capturing an individual’s specific anthropometry and force-generating properties, thus assuring accurate analysis of each individual subject, regardless of the sample size. This represents an improvement in current state-of-the-art modeling methodologies in which the recruited individuals are chosen to have a similar build as that of the musculoskeletal geometry model used^40,41^ or the model anthropometric properties are scaled with no identification of subject-specific activation-to-force parameters^42,43^. This study did not validate the neural data-driven model predictions at the single muscle-tendon level due to the underlying constraints in measuring single muscle force in humans. However, we demonstrated the ability of generating muscle-tendon force solutions that explain both movement neural (motor neuron discharges) and mechanical (joint moments) levels. Future work will investigate the impact that the neuro-mechanical constraints we imposed have on the ability of probing unique multi-muscle force solutions.

In conclusion, we proposed a new paradigm for establishing a window into the human central nervous system and into the processes responsible for the translation of neural information into biomechanical function. We showed that we could probe the mechanical forces elicited by α-motor neurons, something central for investigating the role of neural connectivity between motor neuron pools in the control of biological joint function *in vivo* in the intact human. Our approach has broad applicability for decoding mechanical function from different sources of neural information (i.e. from spinal cord^44–46^, nerve^47^, or intramuscular^48^ readings) pioneering the way to neuro-mechanical man-machine interfaces. That is, interfaces that exploit an individual’s complete neuro-mechanical information for device control rather than only the underlying neural signals^46,49,50^. The translation of these methods to patients with orthopedic and neurological conditions^33,46^ creates unprecedented opportunities to study how neuro-mechanical processes are disrupted in individuals with neuromuscular pathologies^33,51^.

## Methods

### Experimental Design

Experiments were conducted in accordance with the declaration of Helsinki. The University Medical Center Göttingen Ethical Committee approved all experimental procedures (Ethikkommission der Universitätsmedizin Göttingen, approval number 01/10/12). Four healthy men (age: 30 ± 1.9 years, weight: 68.3 ± 1.3 kg; height: 184 ± 2.1 cm) volunteered for this investigation after providing signed informed consent.

We developed a subject-specific modeling formulation (Fig. 6) that enables accurate blinded prediction of leg musculoskeletal function as controlled by motor neuron spike trains decoded *in vivo* from the intact human (Fig. 2). First, ground reaction forces (GRFs) and motion capture data were recorded synchronously using an in-ground force plate (Bertec Co., Columbus, OH, USA, 2048 Hz) and a seven-camera system (Qualisys, Göteborg, Sweden, 256 Hz), respectively. A set of 18 retro-reflective markers were placed on each subject’s pelvis and right lower extremity^13^. Data were recorded during one static anatomical pose and during a set of functional trials for determining the right hip, knee, and ankle joints centers of rotation^52^.

Subsequently, a dynamometer (M3, Biodex Medical Systems Inc., Shirley, NY, USA) was used to measure ankle plantar-dorsi flexion angular moments and positions. The dynamometer data were recorded synchronously with HD-EMGs (Fig. 1). These were measured and A/D converted with 12-bit precision using a 256-channel EMG acquisition system (EMG-USB2, OT Bioelettronica, Torino, Italy). A set of three 64-channel and two 32-channel flexible grid electrodes (10-mm inter-sensor distance) were used. The grids incorporated copper tracks on a kapton support and were applied on the skin surface by 1-mm thick double adhesive foam with holes in correspondence of each sensing site. The skin-grid contact was improved via conductive paste. The 64-channel grids were placed on the tibialis anterior, soleus and gastrocnemius medialis muscles. The 32-channel grids were located on the gastrocnemius lateralis and peroneus group (Fig. 3). As previously described, peroneus group EMG information recorded during dorsi-flexion informed computational representations of the peroneus tertius musculotendon unit^13^. EMG information recorded during plantar-flexion informed computational representations of the peroneus brevis and longus musculotendon units. This provided access to EMG information for a total of seven musculotendon units^13^. All recordings were performed in monopolar derivation and adjacent signals were then subtracted to obtain bipolar recordings for subsequent analysis. Interferent HD-EMGs were processed to derive linear envelopes and enable performance comparison with respect to our proposed approach based on motor neuron discharges (Eqs 1–4). In this context, HD-EMGs were averaged across all recorded channels for each muscle. The amplitude offset of the resulting residual signal was removed to achieve zero mean. The resulting signal was high-pass filtered (30 Hz), full-wave rectified, and low-pass filtered (2 Hz) using a zero-phase second-order Butterworth filter. For each subject and muscle, the resulting HD-EMG linear envelopes were normalized with respect to the peak-processed values obtained from the entire set of recorded trials.

Subjects performed series of isometric plantar-dorsi flexion contractions that tracked a monitor-displayed reference trace. This involved reaching pre-defined percentages of maximal dorsi-flexion and plantar-flexion moments starting from the resting condition (Figs 1–2). Three subjects performed tasks spanning different %MVCs including 30, 50, 70 and 90%MVC. Each %MVC condition was performed at three ankle joint arrangements including anatomical position, 10 degrees dorsi-flexion, and 10 degrees plantar-flexion. External attachments were applied to the subject leg to preserve the knee joint angular position at 60 degrees. Each %MVC and ankle angle condition involved four trials. Each trial comprised four sequential phases including: 2s-phase transitioning from resting to dorsi-flexion %MVC, 2s-phase transitioning from dorsi-flexion %MVC to resting condition, and two equivalent phases of plantar-flexion %MVC (Fig. 2). These tasks were designed so that higher %MVCs underlay higher moment slopes (Fig. 2). The fourth subject performed contractions spanning a different %MVC range, including 20, 30, 40, 50, 60%MVC. The joint moment slope for transitioning between resting condition and %MVC was kept constant (10%MVC/s), while the duration of each phase varied proportionally. In this, each trial was designed to preserve a constant moment slope across all %MVC conditions. The variable-slope and constant-slope trials enabled incorporating different muscle contraction strategies in the validation stage.

During standing static and functional trials, both GRFs and marker trajectories were low-pass filtered (6 Hz) with the same fourth-order Butterworth filter. GRFs from the static pose were averaged over a 3-s time window and the resulting vertical component was used to determine each subject’s weight. During the dynamometer trials, the measured moments and angular positions were low pass filtered at 2 Hz. Moments were then averaged over a 5-s time window during which the subjects did not exert force. Averaged data were used to remove the force offset from the recorded moments due to the weight of the subject’s leg and dynamometer mechanical attachments. Dynamometer data were synchronously recorded with HD-EMG. From the dynamometer trials, two datasets were created. One was used to calibrate the neural data-driven musculoskeletal model and the other (fully separated from the first) for validation. The calibration dataset consisted of six trials taken at the lowest %MVC condition for each subject, i.e. 30%MVC trials for subjects 1–3 and 20%MVC trials for subject 4. Calibration datasets included two trials at ankle anatomical, 10 degrees dorsi-flexion, and 10 degrees plantar-flexion positions respectively for each subject. The validation dataset consisted of all remaining trials spanning unseen %MVC condition, i.e. 207 trials in total (see Statistical Analysis Section).

### Motor Neuron Decoding and Spatiotemporal Patterns of Spinal Cord Activity

Multi-muscle HD-EMG spatial sampling was used to access high-dimensional datastreams of muscle fiber electrical activity. Sampled (2048 Hz) HD-EMGs were band-pass filtered (10–500 Hz) and decomposed to extract the discharges of excitation of the innervating spinal motor neurons^53^ using a recently developed blind source separation algorithm based on deconvolution^48^. We report only the activities of motor neurons corresponding to a threshold of 0.9 in the PNR performance metrics^36^. This ensures that all the reported discharge patterns were decoded with high fidelity^36^. Figure 2 shows how the interferent HD-EMG (soleus, 30%MVC, anatomical ankle position) was separated into its neural (motor neuron discharges) and its peripheral component (muscle fibers action potentials).

Discrete single motor neuron spike trains decoded in the control of a muscle were combined together to form the cumulative spike train^48^. The inverse of the time elapsed between each pair of consecutive spikes was used to determine the discharge rate DR, i.e. the number of pulses per second generated by the motor neuron pool:

1 $$D{R}_{n}=\frac{1}{{t}_{n}-{t}_{n-1}}$$

where t_n_ represents the discharge time of the n^th^ spike in the cumulative train. The DR was smoothed using a moving average window of 500 samples. The resulting discharge pattern provides a direct measure of the net firing of the active α-motor neurons innervating in the spinal cord. Therefore, this was mapped onto the rostrocaudal location of ipsilateral (i.e. right leg side) α-motor neuron pools in the lumbo-sacral segments of the human spinal cord (Fig. 3) to provide access to spinal level activity. This was done based on a previously published myotomal chart^54^ describing how individual spinal segments innervate muscles. Reference segmental charts were compiled for all body muscles by combining anatomical and clinical data from six different sources. Functional magnetic reference imaging of cervical and lumbosacral segments has confirmed the anatomical localization of the published segments^55,56^. Detailed information is documented in the previously published procedures^55,56^. This was employed to reconstruct the output pattern of any given spinal segment S_j_ from individual DR patterns:

2 $${S}_{i}=\frac{\sum _{i=1}^{{n}_{j}}{k}_{ij}\cdot D{R}_{i}}{{n}_{j}}$$

where n_j_ is the number of discharge patterns corresponding to the j^th^ spinal segment and k_ij_ is the weighting coefficient of the i^th^ muscle relative to the j^th^ spinal segment, as reported in^54–56^.

The discrete cumulative spike trains from all motor neurons decoded in the control of a specific muscle was converted into continuous neural activation profiles via a critically damped, linear, second-order, differential system^57^. This characterized the activation and deactivation dynamics in response to motor neuron discharges and was expressed in a discrete form using a time history-dependent, infinite impulsive response filter^58^:

3 $$u(t)=\alpha \cdot x(t-d)-{\beta }_{1}\cdot u(t-1)-{\beta }_{2}\cdot u(t-2)$$

where x(t) is the cumulative spike train at time t, u(t) is the neural activation profile, and α, β_1_, β_2_ are the recursive filtering coefficients. These were constrained to obtain a filter positive stable solution and unit gain: β_1_ = C_1_ + C_2_, β_2  _=C_1_ · C_2_, α − β_1_ − β_2  _=1, with −1 < C_1_, C_2_ < 0. The term d is the electromechanical delay. The resulting u(t) was summed to residual EMGs (i.e. signal component not explained by the identified motor unit activity) to recover the total muscle twitch response. We refer to this as to our estimate of the neural drive to muscle. This was the effective neural information used for driving the musculoskeletal modeling formulation.

### Neural Data-driven Musculoskeletal Modeling

First, we used the open-source software OpenSim to scale a generic lower extremity model of the musculoskeletal geometry to match the subject’s anthropometry^59^. The musculoskeletal geometry model had five lower extremity degrees of freedom (DOFs) at the hip (flexion-extension, adduction-abduction, internal-external rotation), knee (flexion-extension) and ankle (plantar-dorsi-flexion), and incorporated a total of seven musculotendon units (MTUs), including soleus, gastrocnemius medialis/lateralis, peroneus longus/brevis/tertius, and tibialis anterior. During the scaling process virtual markers were placed on the generic musculoskeletal geometry model based on the position of the experimental markers from the static pose and the estimated joint centers from the joint functional trials (see the Experimental Design Section). This procedure linearly scaled the geometry model anthropomorphic properties (i.e. anatomical segment length, width, depth, center of mass location, and mass moment of inertia) as well as MTU insertion, origin and MTU-to-bone wrapping points on the basis of the relative distances between experimental markers, estimated joint centers and corresponding virtual markers.

Then, the subject-specific musculoskeletal geometry model was integrated as part of the proposed neural data-driven modeling formulation (Fig. 6), which comprised five main components^34^. The muscle activation component (Fig. 6A) received as input the decoded neural drive to a muscle (Eq. 3) further processed via a nonlinear transfer function to compute the resulting muscle activation:

4 $$a(t)=\frac{{e}^{Au(t)}-1}{{e}^{A}-1}$$

where −3 < A < 0 is the non-linear shape factor, with 0 being a linear relationship. Muscle activation a(t) reflected the ensemble dynamics of all electro-chemical transformations triggered at the muscle fiber level by the motor neuron discharges^34^. The musculotendon kinematics component (Fig. 6B) synthetized the MTU paths defined in the subject-specific scaled geometry model (see Musculoskeletal Geometry Model Scaling Section) into a set of MTU-specific multidimensional cubic B-splines^60^. Each B-spline computed MTU kinematics (i.e. MTU length and moment arms) as a function of input joint knee and ankle angles^60^. The musculotendon dynamics component (Fig. 6C) used muscle activation and MTU length to control a Hill-type muscle model and estimate instantaneous length, contraction velocity, and force in the muscle fibers, as well as strain and force in the series-elastic tendon within each MTU^52,61^. The joint dynamics component (Fig. 6D) computed ankle plantar-dorsi flexion moments as the product of MTU force and associated moment arms from the MTU kinematics block.

The offline model calibration component (Fig. 6E) identified subject-specific parameters that vary non-linearly with individuals’ anthropometries^52,61^. Calibrated parameters in the muscle activation component (Fig. 6A) included the filtering coefficients C_1_, C_2_ (Eq. 3) and the shape factor A (Eq. 4)^58^. These were global parameters that equally applied to all MTUs. Calibrated parameters in the musculotendon dynamics component (Fig. 6C) included two strength coefficients. These varied within (0.5, 1.5) to scale maximal isometric force nominal values^34,52^ for all dorsi-flexing and plantar-flexing MTUs respectively. This enabled preserving physiological force ratios within the two muscle groups while matching an individual’s force generating capacity. MTU-specific tendon slack length and optimal fiber length were adjusted within ± 8% and ± 3% of their initial value to fine tune muscle-tendon force-length-velocity relationships. Parameters initial values were determined as previously described to preserve normalized values between generic and linearly scaled musculoskeletal geometries^34,52^. A simulated annealing algorithm varied parameters to minimize the sum of the mean square differences between the predicted and experimental joint moments calculated over all calibration trials^13,52,61^.

### Statistical Analysis

After discarding flawed trials, validation trials for all subjects involved a total of 207 trials, i.e. 50, 48, 49, and 60 for each subject respectively. Trials were discarded when HD-EMG electrode failure was detected resulting in noise contamination in the recorded data. Similarity between motor-neuron predicted and experimental moments was calculated using the coefficient of determination (R^2^, square of the Pearson product moment correlation coefficient) and the root mean squared error normalized with respect to the root mean squared sum of the corresponding experimental quantity (NRMSE). The 90% confidence interval was estimated for R^2^ and NRMSE using the Chebyshev’s theorem, i.e., expected interval = mean ± 3.16·std. This could be applied with no assumption on the normality of R^2^ and NRMSE distributions.

### Accession codes

Data and code are available upon request.

## Electronic supplementary material

Similarity between predicted and reference joint moments

From motor neuron discharges to whole-muscle mechanical force
