# Supplementary material for: In Vivo Neuromechanics: Decoding Causal Motor Neuron Behavior with Resulting Musculoskeletal Function
Source: Sci Rep. 2017 Oct 18;7:13465. doi: 10.1038/s41598-017-13766-6 (PMC5647446; doi:10.1038/s41598-017-13766-6)
Supplement: Supplementary file 1 — Similarity between predicted and reference joint moments [file 41598_2017_13766_MOESM1_ESM.pdf]

In Vivo Neuromechanics: Decoding Causal Motor Neuron Behavior with Resulting Musculoskeletal Function

Massimo Sartori[1], Utku Ş. Yavuz[2], Dario Farina[3]

[1] Institute of Biomedical Technology and Technical Medicine, Department of Biomechanical Engineering, University of Twente, Enschede, THE NETHERLANDS

[2] Pain Medicine, Department of Anaesthesiology, University Medical Center Göttingen, Georg-August University, Göttingen, GERMANY

[3] Department of Bioengineering, Imperial College London, London, UNITED KINGDOM

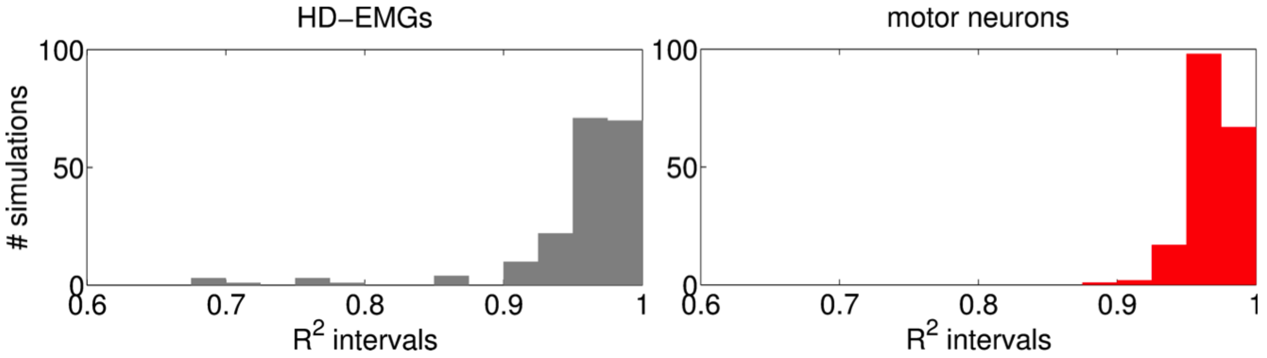

Figure S1: Similarity between predicted and reference joint moments. Distribution of the squared Pearson product moment correlation coefficient (R^2) between reference ankle moment and the moment predicted from motor neuron spike trains as well as using high-density electromyogram (HD-EMG) linear envelopes. Histograms report R^2 distribution across the 207 trials conducted by all subjects and across all angle and percentage maximal voluntary contraction (%MVCs) conditions.
